# Supplementary material for: Breaking the capacity bottleneck of lithium-oxygen batteries through reconceptualizing transport and nucleation kinetics
Source: Nat Commun. 2024 Nov 17;15:9952. doi: 10.1038/s41467-024-54366-z (PMC11570598; doi:10.1038/s41467-024-54366-z)
Supplement: Supplementary file 1 — Supplementary Information [file 41467_2024_54366_MOESM1_ESM.pdf]

Supplementary Information for

Breaking the Capacity Bottleneck of Lithium-Oxygen Batteries through Reconceptualizing Transport and Nucleation Kinetics

Zhuojun Zhang, Xu Xiao*, Aijing Yan, Kai Sun, Jianwen Yu, Peng Tan*

Department of Thermal Science and Energy Engineering, University of Science and Technology of China (USTC), Hefei 230026, Anhui, China

*Corresponding author: xiaoxu@ustc.edu.cn, pengtan@ustc.edu.cn

Multi-physics cross-scale model

The growth of Li_2O_2 particles in the channel is described by the phase field method, which belongs to the microscale simulation. The macroscopic continuum method is used to simulate the mass and electron transport in the electrolyte and on the electrode surface. The two scale methods are connected by an important parameter, the LiO_2 concentration term, transported at the interface of Li_2O_2 particle and electrolyte (solid-liquid interface). The effect of the Li_2O_2 film is manifested by the local failure of the electrochemical surface.

1. Phase field method

In the phase field method, the sharp interface is replaced by the smooth diffusion interface. For the Li_2O_2 particle growth, the diffusion interface is traced by both thermodynamic and kinetic factors. The thermodynamics is referred to the Allen-Cahn equation and the kinetics is determined by the disproportionation reaction of LiO_2. Thus, the governing equation is expressed as

$$\frac{\partial \zeta}{\partial t} = -L\frac{\delta F}{\delta \zeta} - \Gamma \tag{S1}$$

where ζ is the order parameter and is used to depict the independent states of the phases in the system. L is the interfacial migration coefficient. F is the total free energy and the free energy density is expressed as

$$f(\xi, c_i, \phi) = \int_V \left[f_{ch}(\xi, c_i, \phi) + f_{grad}(\nabla\xi, \nabla\phi) + f_{elec}(c_i, \phi) \right] dV \tag{S2}$$

where the Γ is the formation rate of Li_2O_2 via disproportionation reaction, which can be expressed as

$$\Gamma = k_s c_{LiO_2}^2 h'(\xi) \tag{S3}$$

where $h'(\xi)$ is an interpolation function, denoted as $\xi^3(10-15\xi+6\xi^2)$, ensuring the validity at the reaction interface.

2. Continuum method

The transport of oxygen, Li ions, and LiO_2 in the electrolyte is described by the macroscopic continuum method and governed by the mass conservation equation.

$$\frac{\partial(c_i)}{\partial t} = -\nabla \cdot \mathbf{N}_i + S_i \tag{S4}$$

where c_i is the concentration of the species i. **N** is the molar flux, expressed as

$$\mathbf{N}_i = -D_i^{eff} \nabla c_i \tag{S5}$$

Notably, the effective diffusion coefficient (D^{eff}) is affected by the phase separation and is expressed as

$$D^{eff} = D_{liquid}[1 - h'(\xi)] + D_{solid} h'(\xi) \tag{S6}$$

where D_{liquid} and D_{solid} represent the diffusivity of species i in the electrolyte and Li_2O_2 particle, respectively.

S is the source term. For the oxygen and Li ions, it can be expressed as

$$S_i = -\sum_m \frac{as_{i,m}}{nF} j_{m,loc} \tag{S7}$$

where $s_{i,m}$ is the stoichiometric coefficient of species I, and n is the number of electrons that participated in each electrochemical reaction m. In this work, the electrochemical current is contributed by the oxygen reduction via the single-electron pathway. Thus, the $s = n = 1$ for both oxygen and Li ions. Besides, the source term of LiO_2 includes its formation by disproportionation reaction and consumption by disproportionation reaction, which is expressed as

$$S_{LiO_2} = \frac{nj_{loc}}{F} - 2\Gamma \tag{S8}$$

where j_{loc} represents the local current density from oxygen reduction, which is described by the Butler-Volmer equation.

$$\frac{j_{loc}}{nF} = k_{p1} c_{LiO_2} \exp\left(-\eta_c \frac{\alpha nF}{RT}\right) - k_{p2} c_{O_2} c_{Li^+} \exp\left(-\eta_c \frac{\alpha nF}{RT}\right) \tag{S9}$$

where k_p represents the corresponding rate constant and η_c represents the cathodic overpotential.

3. Boundary and initial conditions

Considering the local failure of the electrode surface caused by the Li_2O_2 film, the local current density around the oxygen inlet is set to be 0. According to

$\int i_{loc} dL_{electrode} = I$, the active electrode area will afford a larger local current density. It can be expressed as

$$\int_0^{loss\_site} i_{loc\_loss} dL_{loss} + \int_{active\_site}^{1} i_{loc\_active} dL_{active} = I \qquad \text{(S10)}$$

where I is the applied current density.

The constant concentration of oxygen at the interface of oxygen gas and the electrolyte liquid is set to be 0.5 mM and the constant concentration of Li-ion at the interface of electrode and separator is set to be 0.5 M.

The initial nucleation sites are located at dimensionless L_{pos} = 0.125 and 0.875.

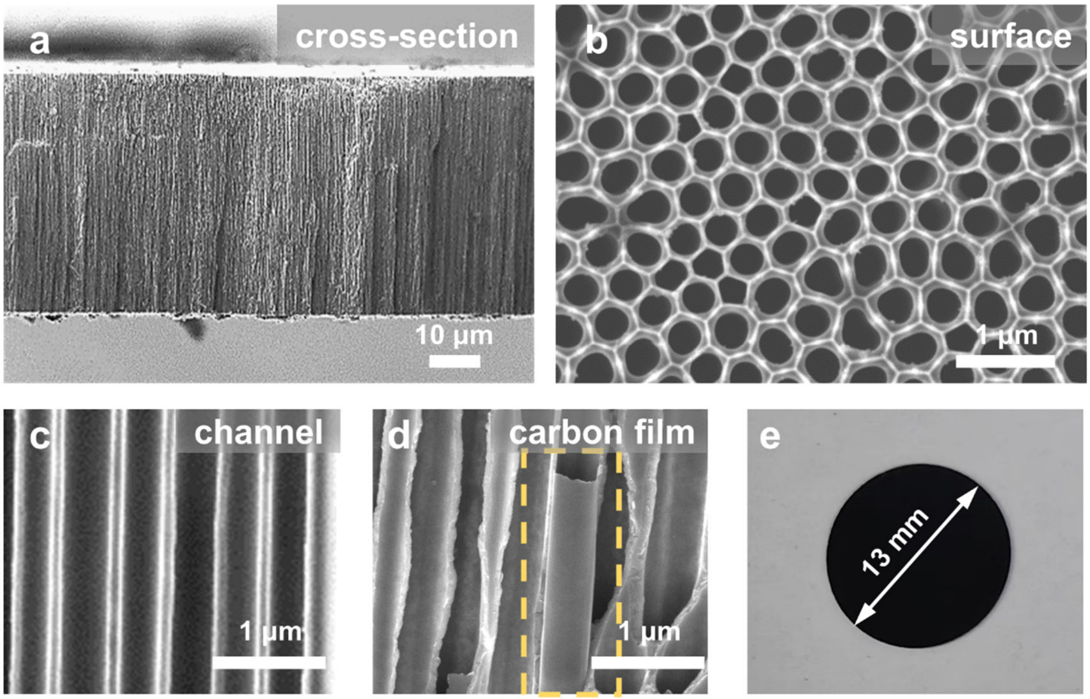

Supplementary Fig. 1. The SEM images and optical photograph of the visualized electrode. **a** Cross-section and (**b**) surface of the electrode. **c** Transport channels through the top and bottom of the electrodes after breaking the electrode off. The diameter of a channel is 390 nm. **d** Carbon film coated on the channel walls supporting electron transport. **e** Photograph of the visualized electrode with a diameter of 13 mm.

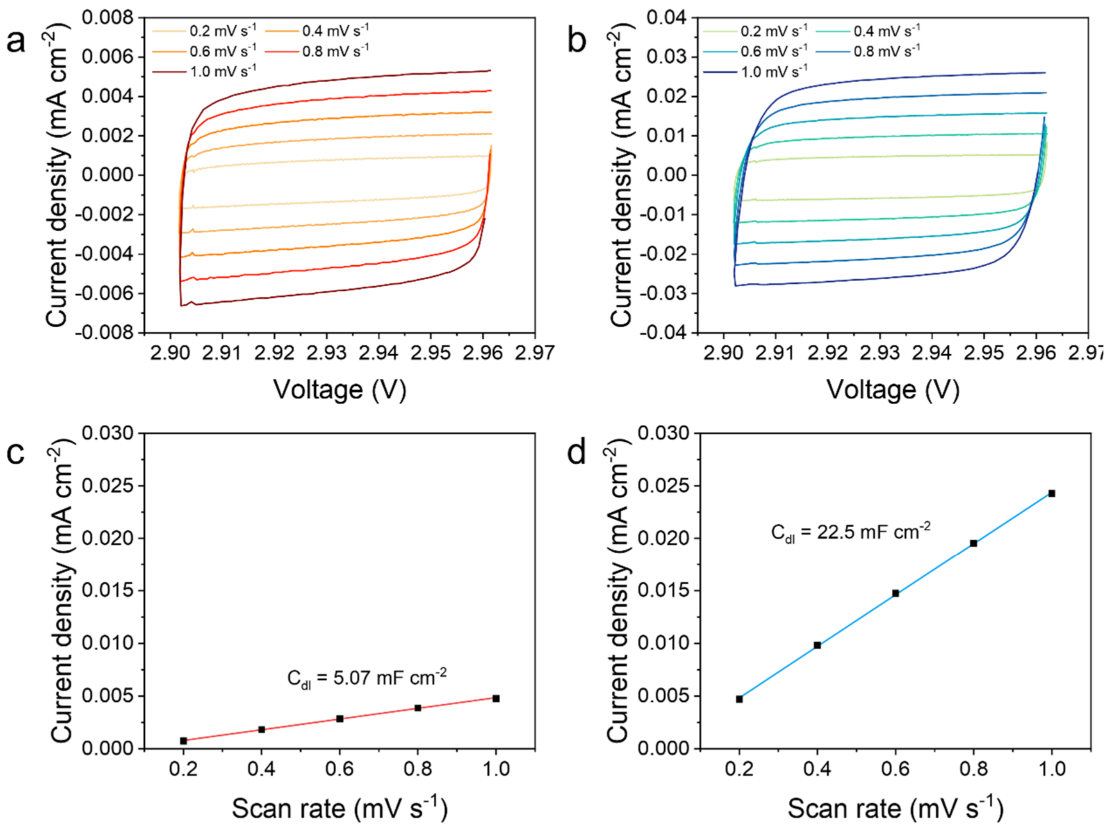

Supplementary Fig. 2. The double-layer capacitance (C_{dl}) of the air electrode measured by a Cyclic Voltammetry (CV) method. CV curves of (**a**) C-AAO electrode and (**b**) CNT electrode in the region of 2.90-2.96 V vs. Li at 0.2-1.0 mV s^{-1} scan rates. C_{dl} of (**c**) C-AAO electrode and (**d**) CNT electrode.

The electrochemical surface area (ECSA) measurements enable more accurate and equitable comparisons of nano-material performance[1]. Since the C_{dl} value is linearly proportional to the electrochemically active surface area of the electrode, the C_{dl} ratio of the visualized (C-AAO) electrode and disordered (CNT) electrode corresponds to their ratio of electrochemically active surface areas. The data points in Supplementary Fig. 2c-d are extracted from the voltage at 2.93 V under various scanning rates in Supplementary Fig. 2a-b. Following fitting, a linear relationship between current density and scanning rates is obtained, the slope of which is the geometric C_{dl}. As a result, $C_{dl,\,CNT}/C_{dl,\,AAO}$ = 22.5/5.07 = 4.44.

To fairly compare the performance of the visualized and disordered electrodes, the current selection is based on the same electrochemically active surface area. The current density of the C-AAO electrode is $i_{areal,\,AAO}$ = 300 mA g^{-1}×0.1 mg /(1.3 cm)^2/3.14×4 = 0.0226 mA cm^{-2}. Thus, the current density of the CNT electrode is $i_{areal,\,CNT}$ = 0.0226 mA cm^{-2}×4.44 = 0.100 mA cm^{-2}.

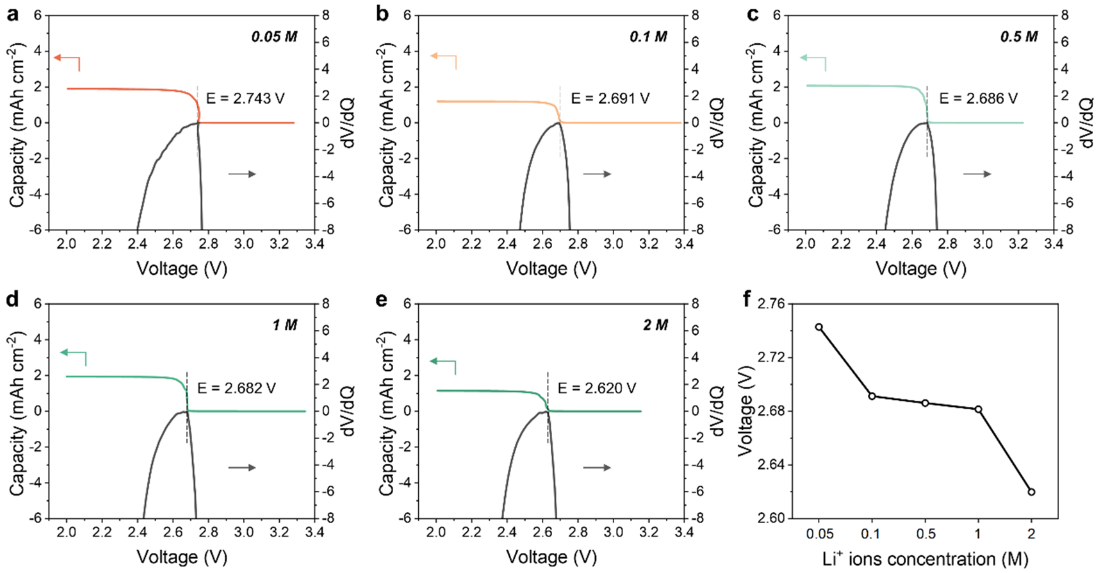

Supplementary Fig. 3. Discharge curves and dV/dQ plots of disordered electrodes with Li^+ ion concentrations of (**a**) 0.05 M, (**b**) 0.1 M, (**c**) 0.5 M, (**d**) 1 M, and (**e**) 2 M. (**f**) The trend of initial voltage plateau with varying Li^+ ion concentrations.

The voltage of the peak points in the dV/dQ plots represent the initial voltage plateau. The initial plateau voltage is decreased with the increasing Li^+ ion concentration.

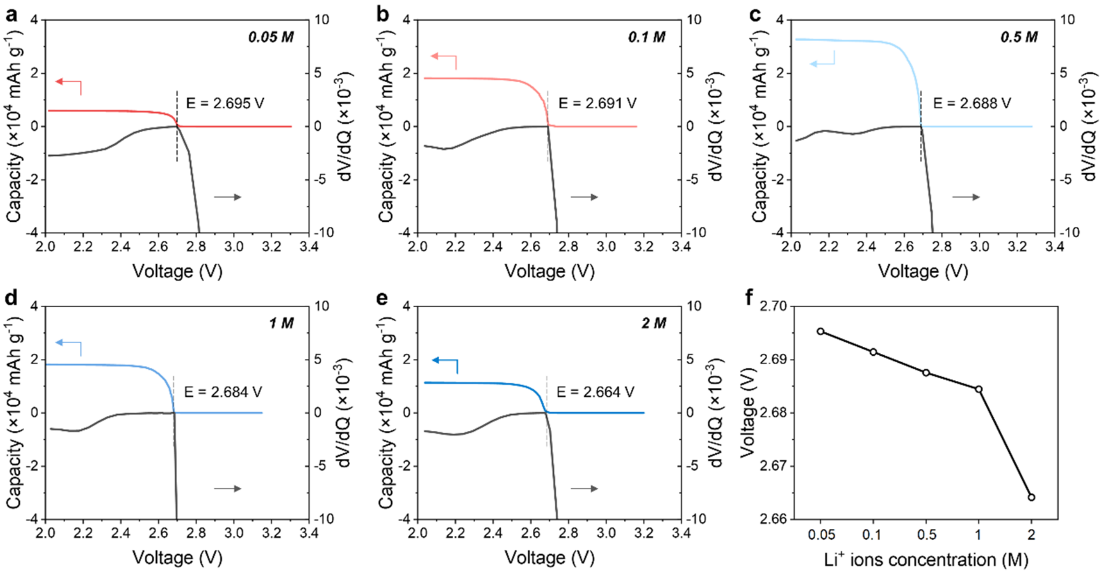

Supplementary Fig. 4. Discharge curves and dV/dQ plots of visualized electrodes with Li^+ ion concentrations of (**a**) 0.05 M, (**b**) 0.1 M, (**c**) 0.5 M, (**d**) 1 M, and (**e**) 2 M. (**f**) The trend of initial voltage plateau with varying Li^+ ion concentrations.

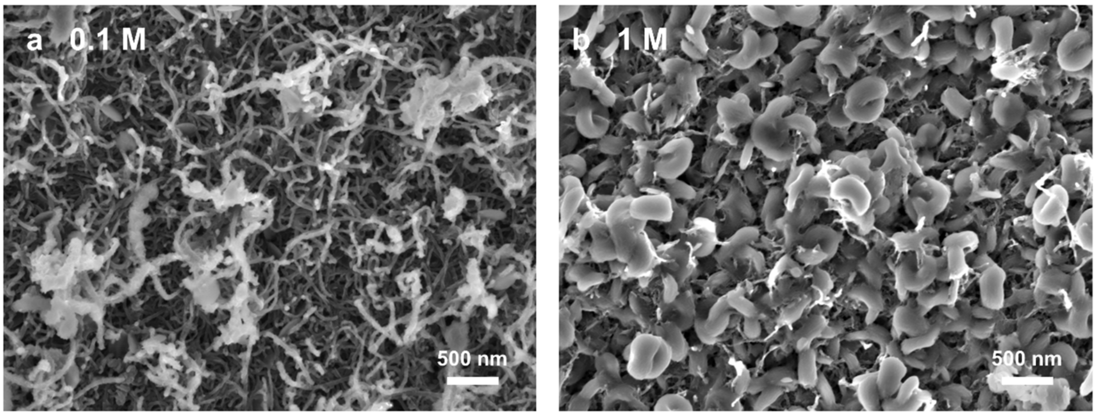

Supplementary Fig. 5. The SEM images of Li_2O_2 morphology on the disordered electrode after full discharge using (**a**) 0.1 M and (**b**) 1M electrolytes.

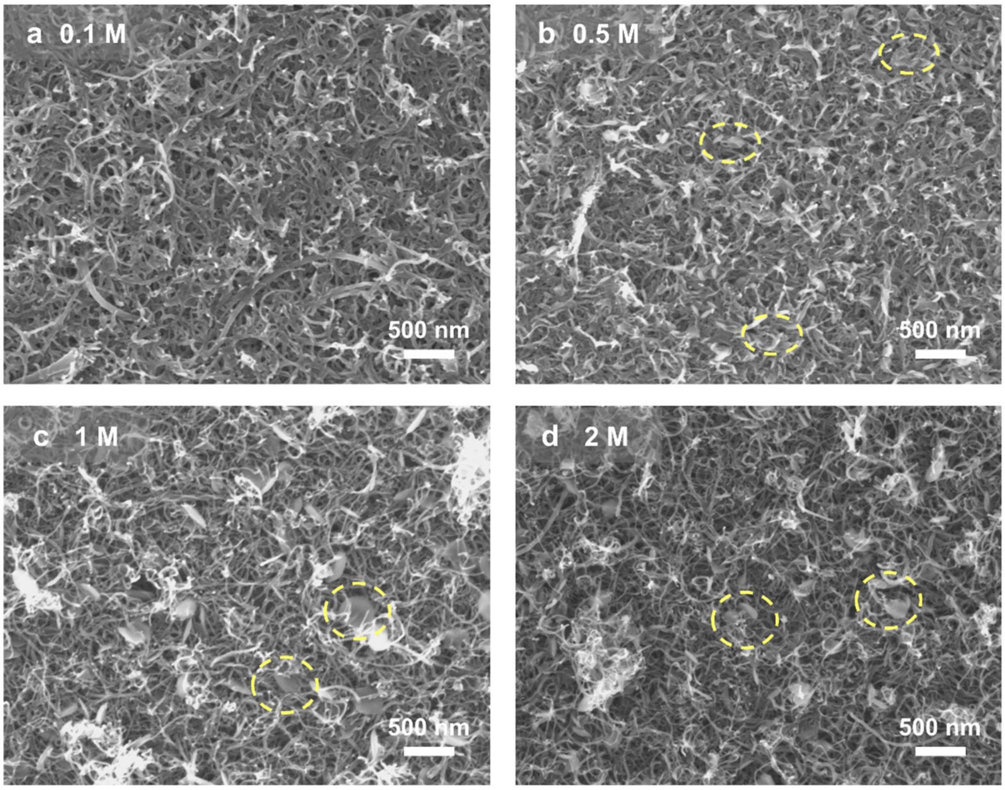

Supplementary Fig. 6. The SEM images of Li_2O_2 morphology on the disordered electrode at the fixed capacity of 1.5 mAh cm^{-2} using (**a**) 0.1 M, (**b**) 0.5 M, (**c**) 1 M, and (**d**) 2 M electrolytes. In the electrolytes with low Li^+ ion concentration (0.1 M), Li_2O_2 appears as a film enveloping the surface of CNTs; while it presents as particles with higher Li^+ ion concentration (0.5-2 M).

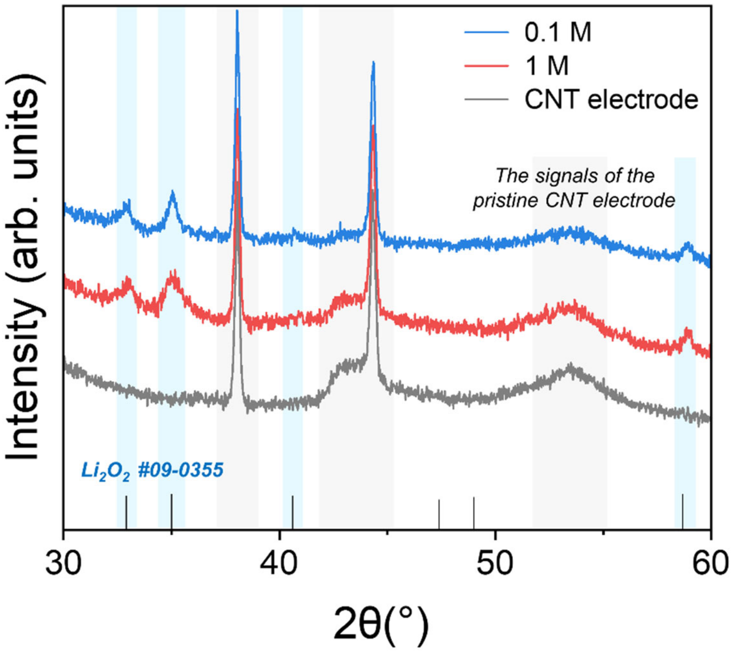

Supplementary Fig. 7. The XRD patterns of the pristine CNT electrode and the CNT electrodes discharged to a fixed capacity of 1.5 mAh cm^{-2} using 0.1 M and 1 M electrolytes. The signals of CNT electrode with 0.1 M electrolyte are weakened more by film-like Li_2O_2.

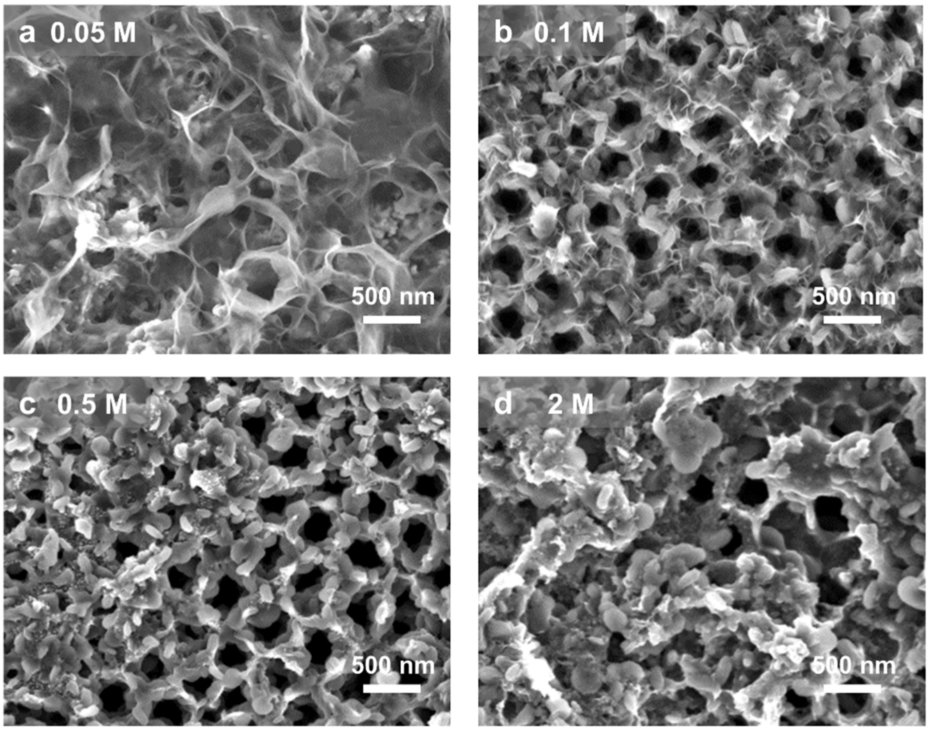

Supplementary Fig. 8. The SEM images of Li_2O_2 morphology on the visualized electrode at the fixed capacity of 4000 mAh g^{-1} using (**a**) 0.05 M, (**b**) 0.1 M, (**c**) 0.5 M, and (**d**) 2 M electrolytes.

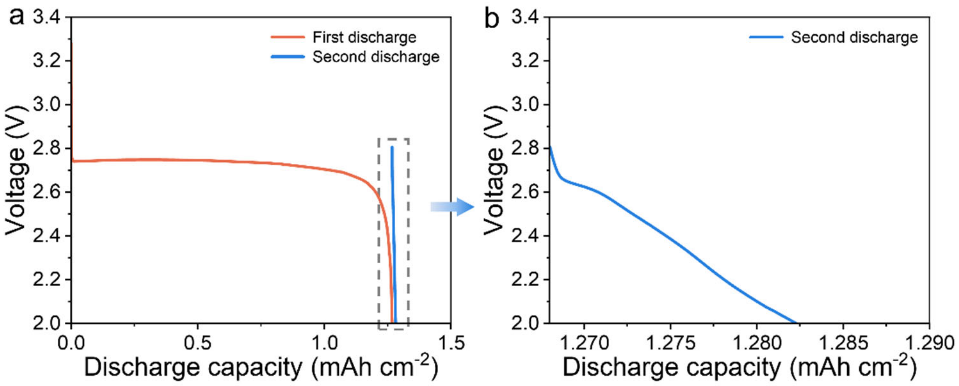

Supplementary Fig. 9. The voltage-capacity curves of the disordered electrode in 0.05 M electrolyte undergoing full discharge, rest, and discharge: (**a**) the first and second discharge. (**b**) Amplification diagram of the second discharge.

A disordered electrode in the 0.05 M electrolyte is fully discharged twice with a current density of 0.1 mA cm^{-2}. Before the second discharge, the battery is rested for 72 hours to regain species concentration at the interface. The negligible capacity of the second discharge implies the predominant contribution of passivating film to the battery failure.

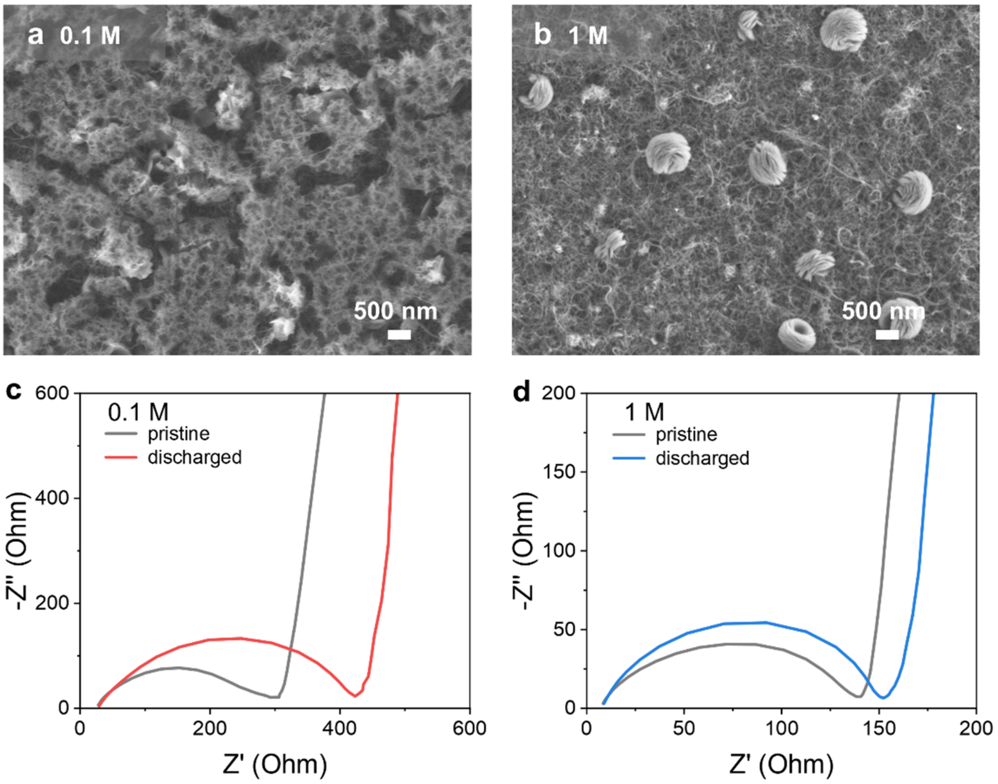

Supplementary Fig. 10. Preliminary investigation on the behaviors of Li_2O_2 and impedance of high-DN electrolyte system. The SEM images of Li_2O_2 morphologies on the disordered electrodes with a fixed capacity of 1.5 mAh cm^{-2} in (**a**) 0.1 M and (**b**) 1 M LiTFSI/DMSO. The corresponding EIS results in (**c**) 0.1 M and (**d**) 1 M LiTFSI/DMSO.

LiTFSI/Dimethyl sulfoxide (DMSO) is used as the high-DN electrolyte. For a fair and direct comparison, all operating conditions of DMSO experiments are the same as the TEGDME experiments. The current density is 0.1 mA cm^{-2} and the fixed capacity is set to be 1.5 mAh cm^{-2}. In the 0.1 M electrolyte, the Li_2O_2 shows a film-like structure, covering the electrode surface. While in the 1 M electrolyte, Li_2O_2 is typically toroidal with the exposed active surface of electrode. Correspondingly, the R_{ct} of 0.1 M electrolyte is notably higher than that of 1 M electrolyte. Both the product morphologies and the EIS trends of the DMSO experiments similar with the TEGDME experiments.

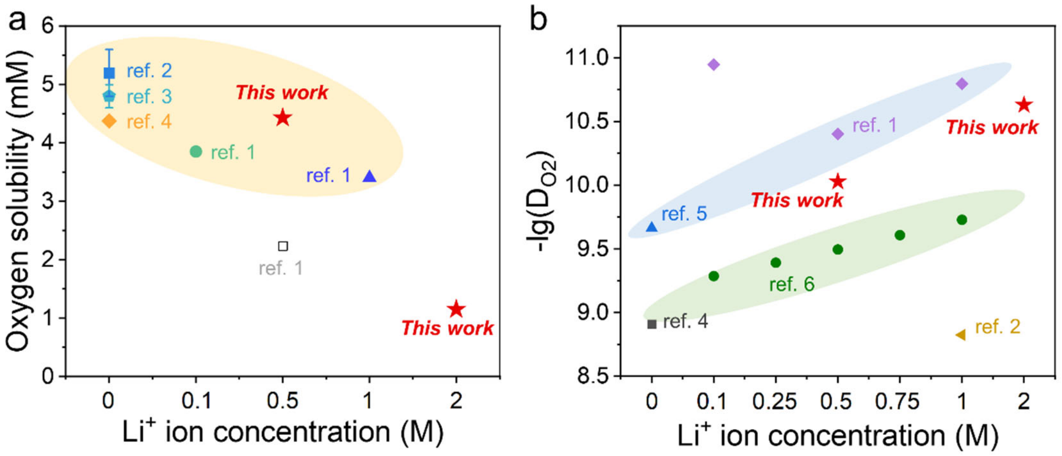

Supplementary Fig. 11. The values of O2 solubility and diffusivity at 1 oxygen pressure measured by experiments and used in the simulation of this work.

Supplementary Fig. 11a shows the oxygen solubility in TEGDME with 0-1 M LiTFSI is at a range of 3.4-5.6 mM under 1 atm oxygen pressure[2–5]. A value of 4.43 mM is selected for the 0.5 M electrolyte. Regrettably, the solubility value for 2 M electrolyte has not been reported. According to the matching degree of simulation and experiment, the solubility is determined to be 1.15 mM in this work. Supplementary Fig. 11b shows the -lg(D_{O2}) from ref. [2,3,5–7], which shows two distinct linear relationship regions. The values of oxygen diffusivity used in the work are selected between the two regions, specifically 9.38×10^{-11} m^2 s^{-1} for 0.5 M electrolyte and 2.35×10^{-11} m^2 s^{-1} for 2 M electrolyte.

The diffusivity of Li^+ ion in the range of 0.5 M-2 M can be expressed by[8]

$$\frac{10^6 D_{Li^+}}{cm^2 s^{-1}} = 1.821c_{Li^+}^2 - 9.051c_{Li^+} + 11.73 \quad \text{(S11)}$$

Thus, the values for the 0.5 M and 2 M electrolytes are 7.66×10^{-10} m^2 s^{-1} and 0.912×10^{-10} m^2 s^{-1}. The above values are summarized in Supplementary Table 4.

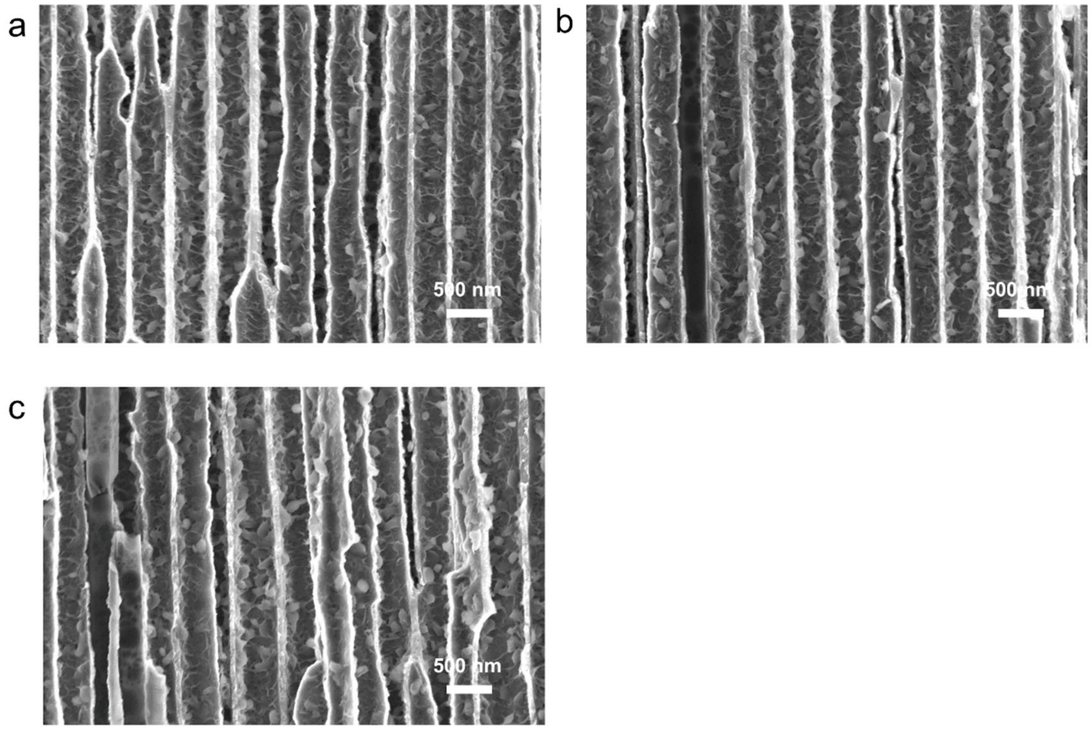

Supplementary Fig. 12. The SEM images of the visualized electrode with 0.05 M electrolyte at (**a**) the oxygen side, (**b**) middle part, and (**c**) separator side.

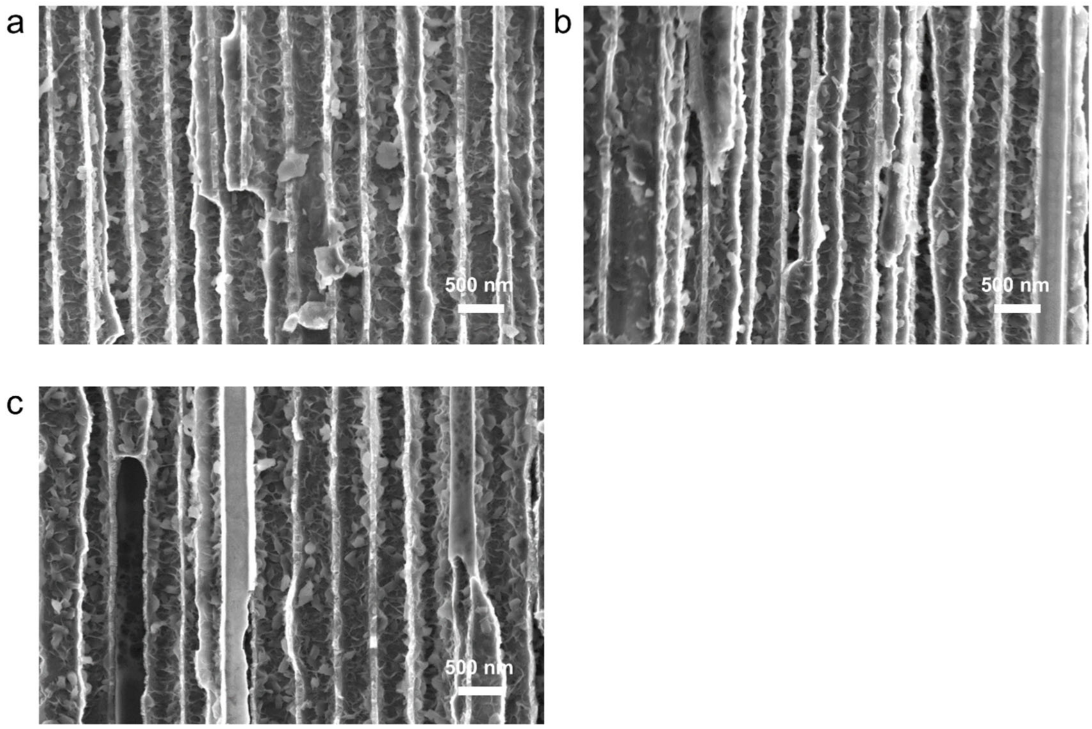

Supplementary Fig. 13. The SEM images of the visualized electrode with 0.1 M electrolyte at (**a**) the oxygen side, (**b**) middle part, and (**c**) separator side.

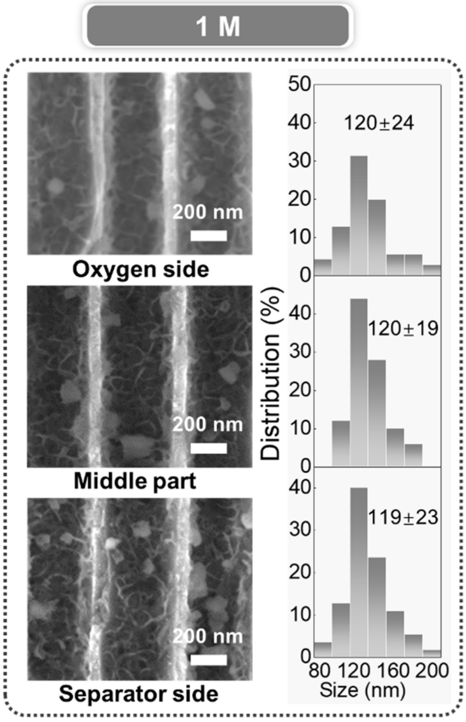

Supplementary Fig. 14. The SEM images of Li_2O_2 distribution at the oxygen side, middle part, and separator side and the statistical analysis of particle size within the 1 M electrolyte.

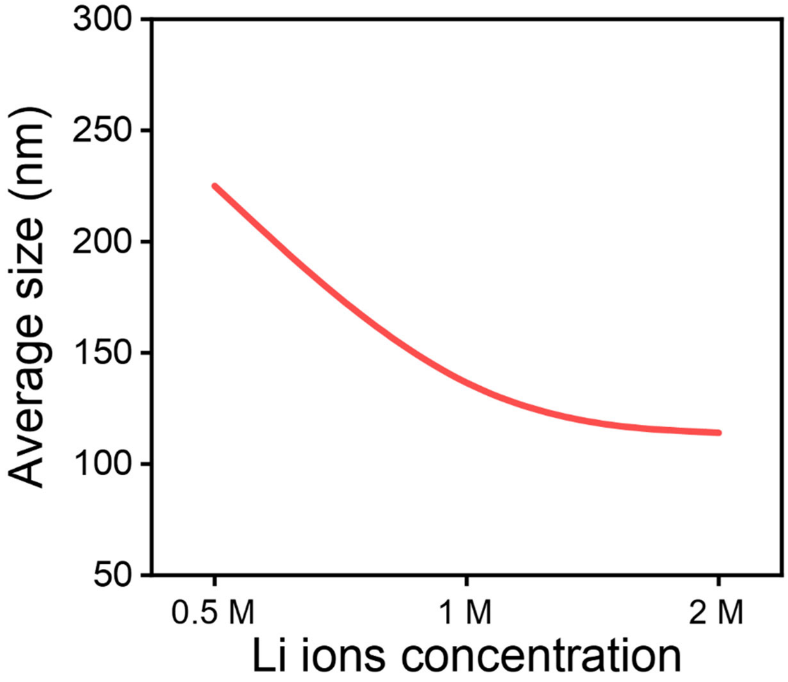

Supplementary Fig. 15. The changing trend of the average size of Li_2O_2 particles in electrolytes with different concentrations.

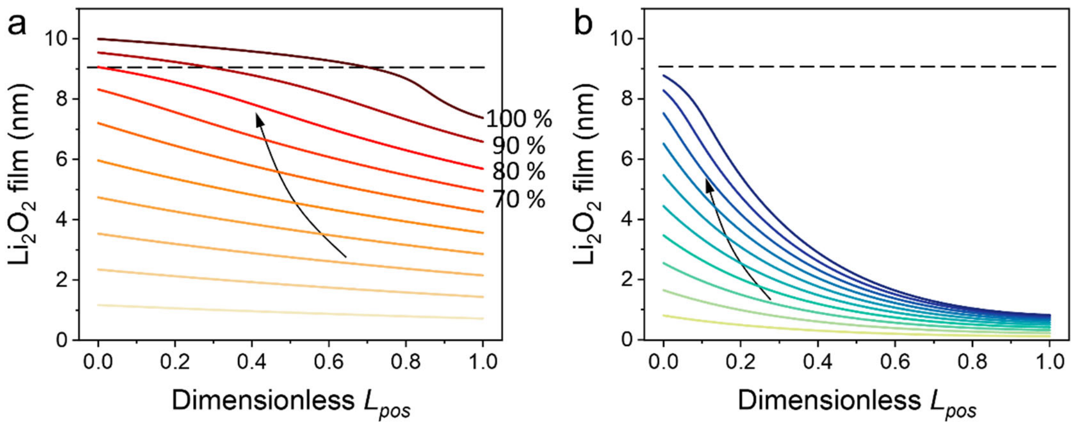

Supplementary Fig. 16. The distribution of the Li_2O_2 film in the electrode depth direction as the discharge proceeds with (**a**) 0.5 M and (**b**) 2M electrolytes.

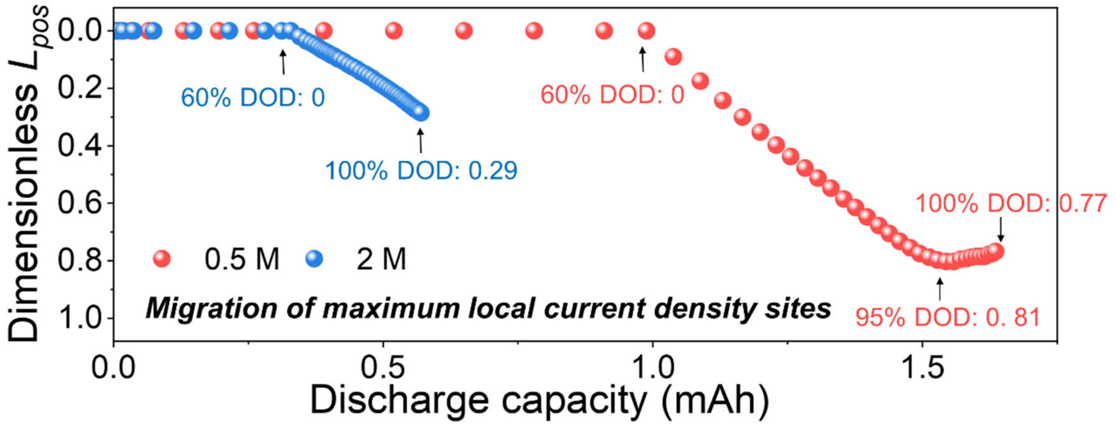

Supplementary Fig. 17. Migration of the peak of the local current density in the electrode depth direction as the discharge proceeds with L_{pos}=0 representing the oxygen face and L_{pos}=1 representing the separator face.

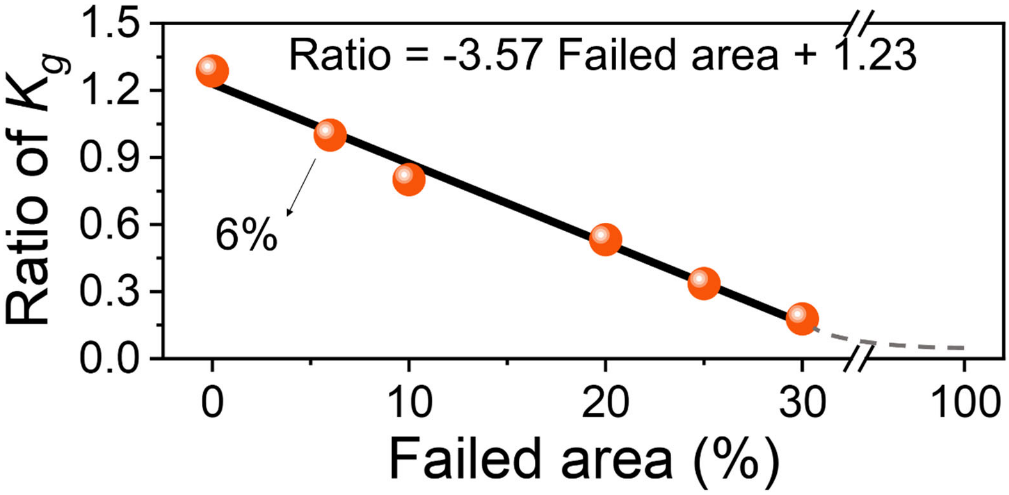

Supplementary Fig. 18. Ratio of growth rate between oxygen side and separator side at the end of discharge under the 0-100 % failed area.

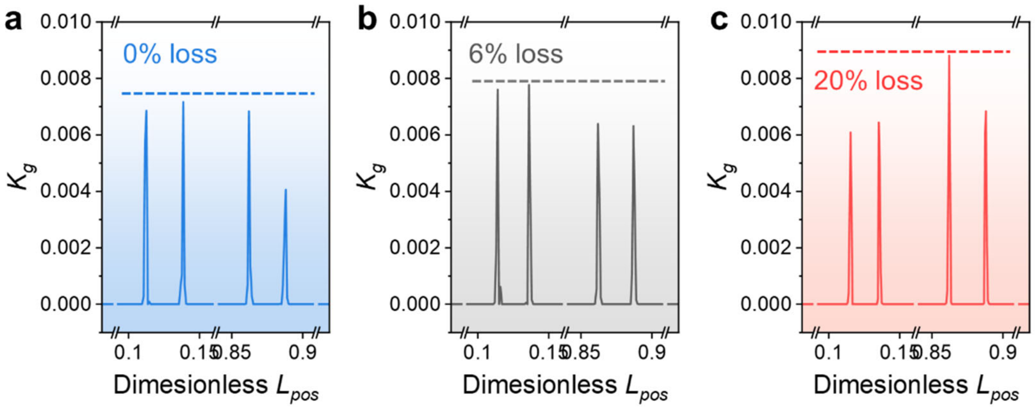

Supplementary Fig. 19. The growth rate of Li_2O_2 particles along the central axis with (**a**) 0%, (**b**) 6%, and (**c**) 20% electrochemical area loss.

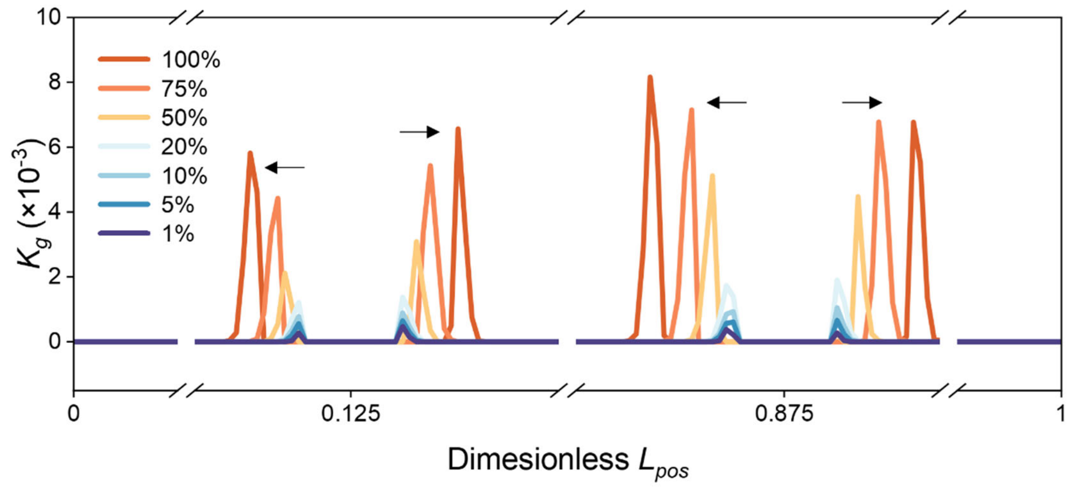

Supplementary Fig. 20. Time-dependent growth rate (k_g) of Li_2O_2 particles along the central axis at 10% electrochemical area loss.

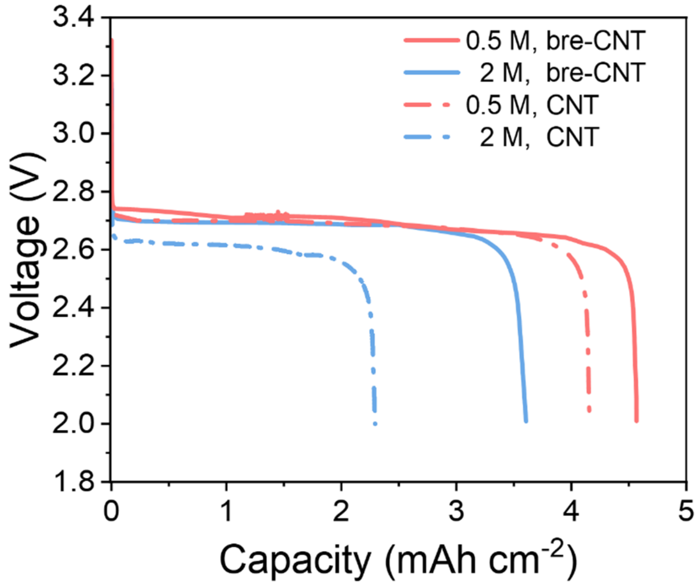

Supplementary Fig. 21. The voltage-capacity curves of CNT and bre-CNT electrodes in the 0.5 M and 2 M electrolytes at 0.1 mA cm^{-2}.

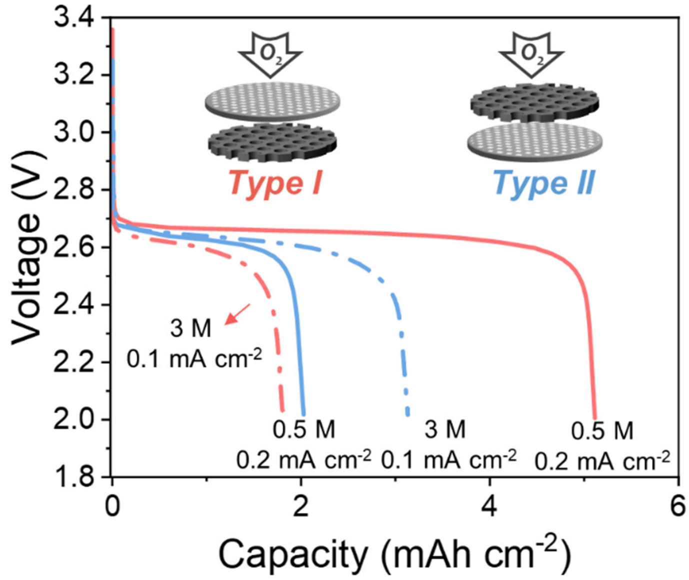

Supplementary Fig. 22. The voltage-capacity curves of type I and type II under the condition of 0.2 mA cm^{-2} and 0.5 M electrolyte and condition of 0.1 mA cm^{-2} and 3 M electrolyte. Red represents structure type I and blue represents type II. Solid lines represent the condition of 0.5 M electrolytes and 0.2 mA cm^{-2}, and dotted lines represent the condition of 2 M electrolytes and 0.1 mA cm^{-2}.

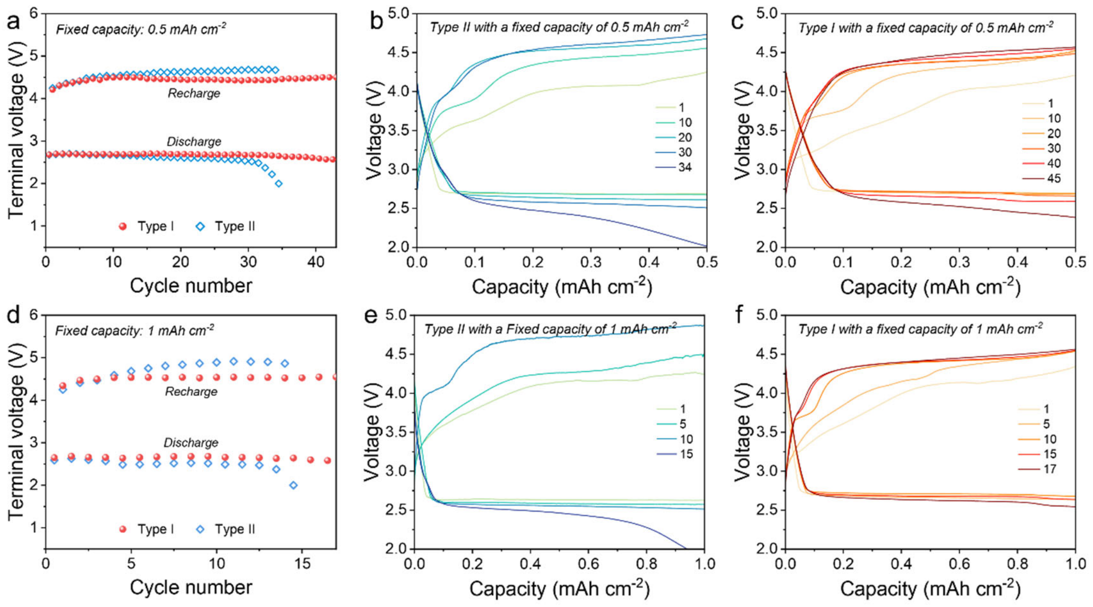

Supplementary Fig. 23. Cycling performance of batteries assembled in type I and type II structures under the current of 0.2 mA cm^{-2} in the 0.5 M electrolytes with the fixed capacities of (**a, b**, **c**) 0.5 mAh cm^{-2} and (**d**, **e**, **f**) 1 mAh cm^{-2}.

A Li-O_2 battery operating under a full discharge-recharge protocol typically exhibits a limited cycle life, as the transport pathways for electrons, ions, and oxygen are significantly disrupted at the end of the discharge. To enhance cycle life, a fixed capacity is usually set during cycling. Operating the battery at a reduced fixed capacity helps preserve the species transport pathways, thereby extending cycle life. Generally, the fixed capacity is set at ~10% of the full discharge capacity[9,10]. Under the same electrode material and electrolyte conditions, achieving a high full discharge capacity means that more oxygen, ion, and electron pathways remain intact during fixed capacity cycling. Therefore, increasing the full discharge capacity is crucial for not only improving the practical capacity (~10% of the full discharge capacity) but also extending the cycling life.

Supplementary Fig. 23a-c shows cycling at a fixed capacity of 0.5 mAh cm^{-2} (corresponding to 10% of the full discharge capacity for type I and 25% for type II), while Supplementary Fig. 23b-d shows cycling at a fixed capacity of 1 mAh cm^{-2} (20% for type I and 50% for type II). Due to the higher full discharge capacity of type I, it exhibits superior cycling stability and potential practical capacity compared to type II.

Supplementary Table 1. The parameters of visualized electrode and CNT electrode

Parameters	Visualized electrode	CNT electrode
Diameter	13 mm	8 mm
Weight	0.1 mg (carbon)	2.5 mg (carbon)
C_{dl}	5.07 mF cm^{-2}	22.5 mF cm^{-2}
Applied current	0.0226 mA cm^{-2} (300 mA g^{-1})	0.1 mA cm^{-2} (20 mA g^{-1})

Supplementary Table 2. The absolute capacities of CNT and C-AAO electrodes using electrolytes with different Li^+ ions concentration

Li^+ ions concentration (mM)	Absolute capacity (mAh)	
	CNT electrode	C-AAO electrode
0.05 M	0.63	0.60
0.1 M	1.20	1.81
0.5 M	2.08	3.27
1 M	1.79	2.61
2 M	1.15	1.14

Supplementary Table 3. The values of R_{ct} and R_s of CNT and C-AAO electrodes at a fixed capacity

Type	State	Symbol (Ohm)	0.1 M	0.5 M	1 M	2 M
CNT electrode	Pristine	R_{ct}	226.4	192.9	184.8	88.13
		R_s	92.89	26.80	17.03	26.55
	Discharged	R_{ct}	350.4	257.8	234.8	134.1
		R_s	121.7	27.59	17.45	29.98
	Net	R_{ct}	124.0	64.90	50.00	45.97
		R_s	28.57	0.790	0.420	3.430
C-AAO electrode	Pristine	R_{ct}	743.1	525.4	556.0	450.5
		R_s	128.1	19.68	21.93	19.80
	Discharged	R_{ct}	1086	611.1	630.6	535.5
		R_s	150.3	21.27	22.16	20.81
	Net	R_{ct}	342.9	85.70	74.60	85.00
		R_s	22.20	1.590	0.230	1.010
Equivalent Circuit	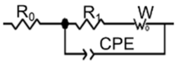					

Supplementary Table 4. The kinetics parameters under different Li^+ ion concentrations used in this work

Li^+ ion concentration (M)	Oxygen solubility (mM)	Oxygen diffusivity (m^2 s^{-1})	Li^+ ion diffusivity (m^2 s^{-1})
0.5	4.43	9.38×10^{-11}	7.66×10^{-10}
2	1.15	2.35×10^{-11}	9.12×10^{-11}

Supplementary Table 5. The used parameters in the multi-physics cross-scale simulation

Parameters	Symbol	Value	Unit	Ref.
Anodic exchange current density	$i_{0,a}$	1	A m^{-2}	[11]
Symmetry factor	α	0.5	1	[11]
Porosity of the separator	ε_{sep}	0.87	1	
LiO_2 diffusivity in the electrolyte	D_{LiO2}	1.54×10^{-10}	m^2 s^{-1}	[2]
Diffusivity in Li_2O_2 particle	D_{solid}	0	m^2 s^{-1}	fitted
O_2 concentration	c_{O_2}	0.5	mol m^{-3}	
Li^+ ion concentration	c_{Li^+}	500	mol m^{-3}	
Anodic rate constant	k_{p1}	2.26×10^{-12}	m s^{-1}	fitted
Cathodic rate constant	k_{p2}	2.26×10^{-10}	m^4 s^{-1} mol^{-1}	fitted
Equilibrium potential	E_c^{eq}	2.96	V	
Initial porosity	ε_0	1	1	
Disproportionation rate constant	k_s	7.22×10^{-8}	m^4 s^{-1} mol^{-1}	fitted

Supplementary References

1. Voiry, D. *et al.* Best Practices for Reporting Electrocatalytic Performance of Nanomaterials. *ACS Nano* **12**, 9635–9638 (2018).
2. Gittleson, F. S., Jones, R. E., Ward, D. K. & Foster, M. E. Oxygen solubility and transport in Li-air battery electrolytes: Establishing criteria and strategies for electrolyte design. *Energy Environ. Sci.* **10**, 1167–1179 (2017).
3. Haas, R. *et al.* Understanding the Transport of Atmospheric Gases in Liquid Electrolytes for Lithium–Air Batteries. *J. Electrochem. Soc.* **168**, 070504 (2021).
4. Wijaya, O. *et al.* A gamma fluorinated ether as an additive for enhanced oxygen activity in Li-O_2 batteries. *J. Mater. Chem. A* **3**, 19061–19067 (2015).
5. Schürmann, A. *et al.* Diffusivity and Solubility of Oxygen in Solvents for Metal/Oxygen Batteries: A Combined Theoretical and Experimental Study. *J. Electrochem. Soc.* **165**, A3095–A3099 (2018).
6. Laoire, C. O., Mukerjee, S., Abraham, K. M., Plichta, E. J. & Hendrickson, M. A. Influence of nonaqueous solvents on the electrochemistry of oxygen in the rechargeable lithium-air battery. *J. Phys. Chem. C* **114**, 9178–9186 (2010).
7. Mohazabrad, F., Wang, F. & Li, X. Experimental Studies of Salt Concentration in Electrolyte on the Performance of Li-O_2 Batteries at Various Current Densities. *J. Electrochem. Soc.* **163**, A2623–A2627 (2016).
8. Mehta, M. R., Knudsen, K. B., Bennett, W. R., McCloskey, B. D. & Lawson, J. W. Li-O_2 batteries for high specific power applications: A multiphysics simulation study for a single discharge. *J. Power Sources* **484**, 229261 (2021).
9. Chi, X. *et al.* A highly stable and flexible zeolite electrolyte solid-state Li–air battery. *Nature* **592**, 551–557 (2021).
10. Kondori, A. *et al.* A room temperature rechargeable Li_2O-based lithium-air battery enabled by a solid electrolyte. *Science.* **379**, 499–505 (2023).
11. Sahapatsombut, U., Cheng Hua & Scott, K. Modelling the micro-macro homogeneous cycling behaviour of a lithium-air battery. *J. Power Sources* **227**, 243–253 (2013).
