# Supplementary material for: Breaking the capacity bottleneck of lithium-oxygen batteries through reconceptualizing transport and nucleation kinetics
Source: Nat Commun. 2024 Nov 17;15:9952. doi: 10.1038/s41467-024-54366-z (PMC11570598; doi:10.1038/s41467-024-54366-z)
Supplement: Supplementary file 2 — Description of Additional Supplementary Files [file 41467_2024_54366_MOESM2_ESM.pdf]

Description of Additional Supplementary Files

File Name: Supplementary Movie 1

Description: The evolution of the volume fraction of Li2O2 particle, thickness of Li2O2 film, oxygen concentration, and local current density during discharge in the 0.5 M electrolyte.

File Name: Supplementary Movie 2

Description: The evolution of the volume fraction of Li2O2 particle, thickness of Li2O2 film, oxygen concentration, and local current density during discharge in the 2 M electrolyte.

File Name: Supplementary Movie 3

Description: The evolution of the Li2O2 growth and LiO2 concentration.
